# Highly Abundant Small Interfering RNAs Derived from a Satellite RNA Contribute to Symptom Attenuation by Binding Helper Virus-Encoded RNA Silencing Suppressors

**DOI:** 10.3389/fpls.2016.00692

**Published:** 2016-05-20

**Authors:** Ricardo Flores

**Affiliations:** Departamento de Virología Molecular y Evolutiva de Plantas, Instituto de Biología Molecular y Celular de Plantas, Universidad Politécnica de Valencia-Consejo Superior de Investigaciones CientíficasValencia, Spain

**Keywords:** satellite RNAs, small interfering RNAs, viral suppressors RNA silencing, micro RNAs, RNA interference

Satellite RNAs (satRNAs) are always found accompanying a helper virus (HV) upon which they are functionally dependent for replication (catalyzed by an RNA replicase coded in part by the HV) and transmission (following encapsidation by the HV coat protein) (Palukaitis, 2016). Most satRNAs have a small size, lower than 700 nt, and do not encode any protein, with the Y satRNA (Y-Sat) of cucumber mosaic virus (CMV) being a representative example. Some small satRNAs attenuate the symptoms incited by their HV, a property presumably resulting from their parasitic behavior. The underlying mechanism, however, could be more complex, specially considering that symptom attenuation by certain satRNAs does not reduce accumulation of the HV.

Such mechanism could be mediated by RNA silencing, which plays key roles in regulating gene expression, maintenance of genome stability and, particularly pertinent in the present context, defense against invading agents including RNA viruses and subviral RNAs (Carthew and Sontheimer, 2009). RNA silencing is triggered by double-stranded (ds) RNAs or highly-structured single-stranded (ss) RNAs that are recognized and processed by Dicer enzymes (Dicer-like, DCL, in plants) into small RNAs (sRNAs): micro RNAs (miRNAs, 21–22 nt) of endogenous origin, and small interfering RNAs (siRNAs, 21, 22, and 24 nt) of endogenous or external origin. The miRNAs and siRNAs are then transferred to Argonaute (AGO) proteins that form the core of the RNA inducing silencing complex (RISC) and guide it to inactivate, by cleavage or translational arrest, their complementary RNA. There is firm evidence supporting that RNA silencing targets plant RNA viruses, prominent among which is their ability to counteract this mechanism by encoding viral suppressors of RNA silencing (VSRs) (Csorba et al., 2015). Many VSRs bind sRNAs and impede their load into RISC, precluding inactivation of viral RNAs by the cognate siRNAs, or disrupting miRNA-mediated developmental stages (eventually resulting in symptoms).

Shen et al. (2015) now report experiments aimed at investigating how Y-Sat impacts on the ability of two VSRs, the CMV-encoded 2b and a tombusvirus-encoded p19, to suppress gene silencing in *Nicotiana benthamiana* induced by self-complementary hairpin RNA (hpRNA). First they observed that Y-Sat attenuates the severe symptoms incited by the Fny strain of CMV, the VSR 2b of which is a strong virulence player. This finding led them to consider that Y-Sat could interfere with 2b function, an idea consistent with the extreme abundance of Y-Sat siRNAs in infected tissues when compared with that of siRNAs from the three CMV genomic RNAs. To test this hypothesis, 2b-expressing constructs were agroinfiltrated into *N. benthamiana* plants infected with Q-CMV, Q-CMV plus Y-Sat, or just mock-inoculated, with the hpRNA-induced silencing of the β-glucuronidase (GUS) reporter gene being measured in the leaves. The Q strain of CMV was used because, despite supporting high Y-Sat replication and accumulation of Y-Sat siRNAs, it encodes a weak 2b VSR with a negligible activity compared with that of two agroinfiltrated strong VSRs: the 2b from another CMV strain and the tombusvirus p19. When GUS and hpGUS constructs were co-infiltrated with 2b, the GUS activity was mostly restored in the mock-inoculated and CMV-infected plants, but not in those co-infected with CMV and Y-Sat, thus indicating that Y-Sat interferes with 2b function. Parallel experiments, replacing 2b by p19, led to conclude that Y-Sat may interfere with the functions of the two VSRs through a similar mechanism. Moreover, in a more complex experimental setting, Y-Sat infection reduced the expression of five miRNA target genes, suggesting that suppression of miRNA function by 2b (in conjunction with another VSR) was overcome by Y-Sat.

Regarding the mechanism involved, RNA immunoprecipitation (with a p19 antibody) and Northern-blot analyses indicated that p19 suppresses the silencing of GUS expression by sequestering the hpGUS-derived siRNAs, which were outcompeted (turning them available for directing GUS silencing) by the abundant Y-Sat-derived siRNAs in Q-CMV plus Y-Sat-infected tissues. Unfortunately, the lack of an antibody against 2b precluded performing a similar experiment with this VSR, which presumably operates through the same mechanism given that it suppresses RNA silencing by binding sRNAs (Duan et al., 2012; González et al., 2012).

The present study (Shen et al., 2015) bears a relationship with a previous one showing that the abundant siRNAs derived from defective interfering RNAs associated with a tombusvirus can saturate its p19 VSR, thus increasing the levels of free viral siRNAs and, concomitantly, reducing the virus accumulation and symptom severity (Havelda et al., 2005). Similarly, the highly abundant Y-Sat-derived siRNAs would saturate the 2b VSR, minimizing its ability to disrupt developmental pathways regulated by host sRNAs and eventually attenuating symptoms. However, this conclusion is mostly based on transient assays, which differ from the natural context.

Ironically, RNA silencing not only mediates symptom attenuation by Y-Sat, but also the yellowing induced in *Nicotiana* spp. Compelling evidence supports that a 22-nt siRNA derived from the Y-Sat determinant associated with symptoms, targets for cleavage the mRNA from the chlorophyll biosynthetic gene *Chl1* at the complementary site predicted by RNA silencing (Shimura et al., 2011; Smith et al., 2011). Site-directed mutagenesis to make the pathogenic determinant of the infecting Y-Sat complementary to the corresponding 22-nt fragment of the *A. thaliana* and tomato *Chl1* mRNAs results in the leaf yellowing, with the *Chl1* mRNA being also downregulated in transgenic tobacco lines expressing Y-Sat inverted repeats (Shimura et al., 2011). Moreover, transformation of tobacco with an RNA silencing vector targeting *Chl1* mRNA incited Y-Sat-like symptoms, while transformation with a silencing-resistant variant of this gene precluded symptoms induced by Y-Sat infection (Smith et al., 2011). A scenario to explain cleavage of the *Chl1* mRNA triggered by AGO1 loaded with the 22-nt siRNA derived from Y-Sat has been proposed (Shimura et al., 2011); this scenario is particularly attractive considering that AGO1 loads siRNAs with a 5′ U, and that the 22-nt siRNA derived from Y-Sat has four Us at its 5′ terminus. In summary, Y-Sat-derived siRNAs play two roles: by saturating the VRS they attenuate symptoms, and by (some of them) specifically loading AGO1 they promote cleavage of an endogenous mRNA and induce disease.

## Author contributions

The author confirms being the sole contributor of this work and approved it for publication.

### Conflict of interest statement

The author declares that the research was conducted in the absence of any commercial or financial relationships that could be construed as a potential conflict of interest.
